# Short Dysfunctional Telomere Is Highly Predictive of Dismal Outcome in MDS but Not in AML Patients

**DOI:** 10.18502/ijhoscr.v14i3.3728

**Published:** 2020-07-01

**Authors:** Nadia El Menshawy, Shaimaa El Ashwah, Mohamed A. Ebrahim

**Affiliations:** 1 Clinical Pathology, Hematology Unit, Faculty of Medicine, Mansoura University, Mansoura, Egypt; 2 Clinical Hematology Unit, Internal Medicine Department, Faculty of Medicine, Mansoura University, Mansoura, Egypt; 3 Medical Oncology Unit, Internal Medicine Department, Faculty of Medicine, Mansoura University, Mansoura, Egypt

**Keywords:** Relative telomere length, Myelodysplatic syndromes, Acute myeloid leukemia

## Abstract

**Background:** A trigger for initiation the clonal hematopoietic stem cells disorders could be short telomere length probably due to chromosomal instability. The relationship between relative telomere length (RTL) and the two linked hematological stem cell disorders, myelodysplastic syndromes (MDS) and acute myeloid leukemia (AML) is still unclear.

**Materials and Methods:** We evaluated the role of RTL in MDS (n=96) and AML (n=130) at the time of diagnosis using a real time quantitative polymerase chain reaction (RT-PCR) technique. The median value of RTL (1) was set as the cutoff for statistical comparison. Overall survival (OS) is defined as the time from diagnosis to death or last follow-up.

**Results: **RTL was significantly longer in both MDS and AML cases versus control (p<0.0001) and was significantly longer in MDS versus AML cases (p =0.03). RTL correlated negatively with age in MDS (p <0.0001) but not in AML cases. RTL was also significantly shorter in MDS cases with pancytopenia and poor risk cytogenetics (p < 0.0001 for each) and short RTL was significantly associated with inferior survival (p = 0.007), while RTL showed no significant impact on OS in AML cases. Moreover, short RTL retained independent prognostic value in multivariate analysis (HR= 3.42 [95% CI, 8.97-19.35], p = 0.004).

**Conclusion: **RTL showed an association with both AML and MDS; however, short RTL was an independent poor prognostic factor in MDS patients only.

## Introduction

 MDS are clonal disorders originating from hematopoietic stem cells, occurred mainly in elderly patients, characterized by ineffective hematopoiesis and a high risk of progression to AML with poor prognosis and inferior outcome^   1 ^. The revised initial International Prognostic Scoring System (IPSS–R) is based on cytogenetic, cytopenia and blast count risk categories ^2^^, ^^3^ .

More than 75 % of MDS cases carry > 1 somatic mutation. These mutations are likely responsible for the different MDS subtypes and epigenetic changes e.g. *TET2, DNMT3A, IDH1/2*, which direct chromatin remodeling, signaling molecules e.g. *NRAS, JAK2, NPM1, FLT3* and the checkpoint regulator *TP53*^   4 ^.

Various researches have demonstrated that there is an overlap of the same genetic mutations seen in MDS/AML patients and the normal adult population that show increasing frequency with age^        5 ^, and until now they still did not know what drives these clones to further genomic instability and the development of MDS/AML.

Telomeres are terminal nucleoprotein structures that entails hundreds to thousands of repetitive sequences of TTAGGG. Organized with the shelter in complex, form a cap to protect the chromosomal ends from degradation and DNA repair responses^   6 ^. Repair of critically short telomeres by telomerase is limited in most somatic cells and cellular senescence is elicited when too many short telomeres accumulate^   7 ^.That is why short telomere provides a driver for human cell apoptosis and has antitumor mechanism. The variation in telomere length between individuals is partly explained by age, and genetic variation^        8 ^**.**

Telomere shortening is countervailed by the telomerase complex, inactive in most somatic cells, but activated in stem cells, including hematopoietic stem cells and cells of most of human cancers^   9 ^. This gives these cells the capability to sustain telomere length^   10 ^, escape senescence and cessation of cell proliferation that occurs when the telomer length reaches a crucial level^   11 ^.

It has been reported that MDS cases with short telomeres-length were more prone to leukemic transformation^        12 ^. Gadji Adebayo Awe^13^ proposed that telomere dysfunction reinforces the chromosomal alterations involved in MDS progression to AML and de novo AML. In a preclinical study, telomere dysfunction produced DNA damage similar to that associated with known MDS phenotypes, leading to altered differentiation in myeloid progenitor cells^        14 ^.

In this study, we measured leukocyte relative telomere length (RTL) in patients with MDS and AML clarifying its clinical and prognostic implications.

## MATERIALS AND METHODS

**Patients**

This study was carried on 96 MDS patients (51 males, 45 females), 130 de novo AML patients (69 males, 61 females) recruited at oncology Mansoura university center from April 2015-untill April 2018, in addition to 50 healthy subject matched in age and sex as reference control. Diagnosis of MDS and AML was established according to 2008 WHO diagnostic criteria^        2 ^. The median value of RTL^ 1^ was set as the cutoff for statistical comparison. Overall survival (OS) is defined as the time from diagnosis to death or last follow-up.

Local ethical issues of research were followed by informed consents from every patient. Cases were followed-up for 3 years to assess prognosis and outcome.

Risk stratified management was done for studied cases according to our local institutional protocols. Immediate treatment was initiated for patients with symptomatic cytopenia in MDS. Lenalidomide was prescribed in red blood cell transfusion-dependent patients with deletion 5q MDS AML. MDS cases with excess blasts were treated by induction chemotherapy and followed by allogeneic hematopoietic cell transplantation or consolidation therapy with high-dose cytarabine in case of good risk AML. Relapsed AML cases were salvaged by either HAM or FLAG protocol, then proceeded to allogeneic hematopoietic cell transplantation if not done, while MDS cases were treated with best supportive care.

**Sampling**

Two ml EDTA peripheral-blood samples were obtained for complete blood count (CBC) and DNA extraction. Bone marrow aspirate and bone marrow biopsy specimens were collected from patients for morphologic and immunophenotypic diagnosis. Cytogenetic study by conventional karyotyping and molecular FISH was done in specific accredited hematology lab for international Canadian accreditation.

**DNA extraction**

DNA was extracted using Thermo Scientific Gene JET Whole Blood Genomic DNA Purification kit according to the protocol of manufacturer’s instructions. The extracted DNA was stored frozen at - 20 C. The DNA samples were quantified by Nano-Drop instrument, the samples were measured 17 - 45 ng /μL** .**

**Relative telomere length measurement**

Leukocyte telomere length was measured using a real time quantitative polymerase chain reaction (RT-PCR) technique developed by Cawthon (15)with minor modification. Cawthon method compares signals from telomere repeat copy number (T) to a single-copy gene copy number (S) and allows calculation of a relative T/S ratio. The primer sequences (written 5′→3′) were tel1b: 5_CGGTTTGTTTGGGTTTGGGTTTGGGTTTGGGTTTGGGTT3_; tel2b:5_GGCTTGCCTTACCCTTACCCTTACCCTTACCCTTACCCT-3_; 36B4u: 5′-CAG CAA GTG GGA AGG TGT AAT CC-3′; 36b4d: 5′-CCC ATT CTA TCA TCA ACG GGT ACA A-3′. The final telomere primer concentration was 100 pico mole. Standard curve of known concentration was used. The order of DNAs from cases and controls was randomized on 96-well plate over two runs with duplication of 4 samples all over the runs for quality control purpose. Each PCR well contained DNA (35 ng/aliquot), 10 ul of the SYBR® Green master mix and 1 ul of forward primers, 1 ul of reverse primers specific for each plate T and S, PCR reagents and DNase-free water to reach 20ul/aliquot. PCRs were performed on the ViiA™7 system/ 96-well block (0.2 mL), Software v1.2 (Applied Biosystems). Thermal cycling profile for both amplicons began with 95°C incubation for 10 min to activate the AmpliTaq Gold DNA polymerase. For telomere PCR, there followed 18 cycles of 95°C for 15 s, 54°C for 2 min. For 36B4 PCR, there followed 30 cycles of 95°C for 15 s, 58°C for 1 min. 

Relative telomere length calculation 

Relative T/S values were calculated according to 2^-ΔΔ Ct^

ΔCt= Ct (calibrator) – Ct (unknown sample).

ΔΔCt = ΔCt (telomere) – ΔCt (single copy gene).

**Cytogenetic analysis**

Conventional cytogenetic by G-banding and interface fluorescence in situ hybridization (FISH) on pretreatment bone marrow samples preserved on sodium lithium heparin from all patients were studied using standard techniques, ten or more metaphases were examined in those patients, and chromosomal abnormalities were described according to the International System for Human Cytogenetic Nomenclature (ISCN)^   16 ^. All specimens were also analyzed by FISH using a comprehensive DNA probe set allowing for the detection of the most relevant recurrent chromosomal translocation in AML- and MDS , different Probes for t (15; 17), t (8; 21), inv 16, and 11 q rearrangement for AML cases (Vysis, London, UK). Analysis of at least 100 metaphase for every case through cell images were captured using a CCD camera (Photometrics SenSys camera) and CytoVision system for image analysis (Applied Imaging). Examination of each case was done by an expert and professional highly specialized staff at international Canadian accredited lab.

 **Statistical analysis**

Data were analyzed using IBM-SPSS© for windows version 1‎‏9.0‏‎. A two-sided p value of ‎‏>‏‎ 0.05 was required for statistical significance. The Chi-square ‎test was used for testing the relation between categorical variables. Mann–Whitney U test or Kruskal–Wallis H test were used for comparison between two or more groups. Correlations were ‎identified by Kendall's Tau correlation ‎coefficient. Survival was determined by the ‎Kaplan-Meier test, the Log- rank test was used for comparison. Independent hazards of different prognostic factors were tested by the Cox's regression model.

## Results

 This study was conducted on 96 MDS patients (51 Males, 45 females), with a median age of 55 years (37-76 years), while AML group consisted of 130 cases, of whom 69 were males and 61 were females, with a median age of44 years (17-67 years). Descriptive data of studied patients are illustrated in Table 1.

**Table 1:** Baseline patients characteristic

		**AML**		**MDS**	
		No	%	No	%
Sex	Male	69	53.1%	51	53.1%
	Female	61	46.9%	45	46.9%
Age	Median (range)	44 (17-67)		55 (37-76)	
WBCs x 1000/µL		45.0- (1.7-214)		2.8- (0.9-10.6)	
Hemoglobin (g/dl)		7.5- (5.4-11.2)		6.5- (3.9-12.8)	
Platelets x 1000/µL		63- (10-120)		88- (18-230)	
BM blast percentage		55- (22-90)		12- (3-28)	
Performance status (EGOC)	0	32	24.6%	29	30.5%
	1	42	32.3%	34	35.8%
	2	28	21.5%	21	22.1%
	3	28	21.5%	12	12.6%
Cytogenetics (Karyotype as defined by WHO in MDS)	Favorable (good)	49	37.7%	36	37.5%
	Intermediate	46	35.4%	14	14.6%
	Poor	35	26.9%	46	47.9%
Cytopenia	Monocytopenia			31	32.3%
	Bicytopenia			24	25.0%
	Pancytopenia			41	42.7%
IPSS-R*	Very Low/Low			35	36.5%
	Intermediate			33	34.4%
	High/Very High			28	29.2%
Response to Induction	Treatment Failure	36	27.7%		
	Complete Remission	94	72.3%		
Relapse	Disease Free	85	90.4%		
	Relapsed	9	9.6%		
Mortality	Censored	96	73.8%	44	45.8%
	Died	34	26.2%	52	54.2%

* IPSS-R: Revised-international prognostic scoring system

**Figure 1:**
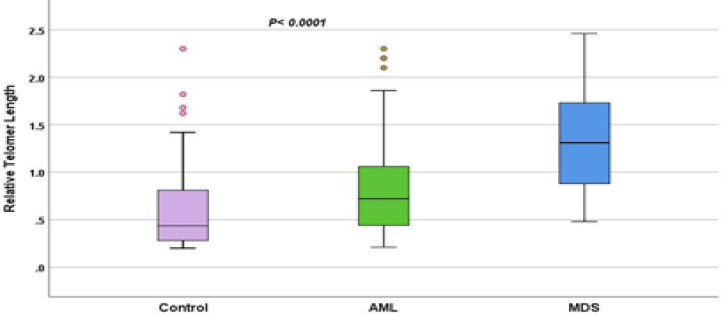
RTL in control, AML and MDS cases

**Fig. 2:**
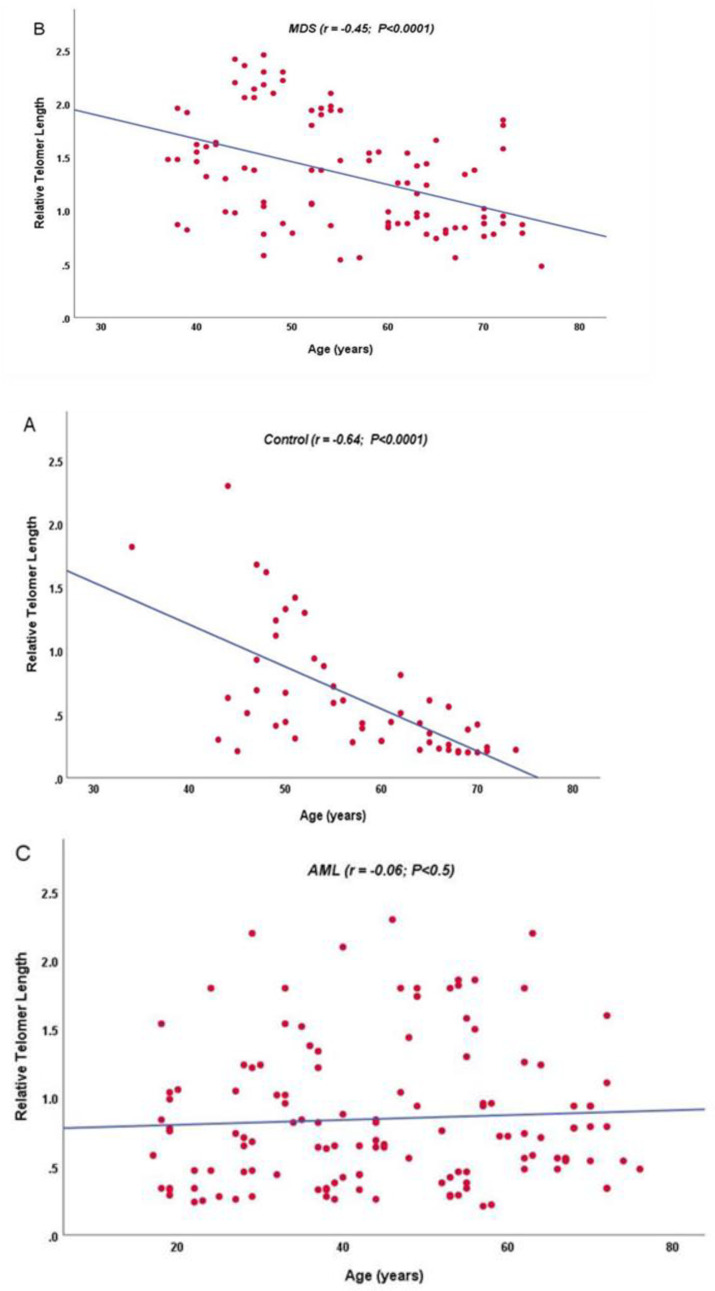
RTL correlates negatively with age in control subjects (A), in MDS patients (B) but not in AML cases (C)

RTL was longer in both MDS and AML cases than control subjects (p < 0.0001) (Figure 1) and was longer in MDS cases than AML cases (p = 0.03). Control and MDS samples revealed significant diminution in telomere length with older age at diagnosis (p < 0.0001), while AML samples showed no significant correlation between RTL and age p = 0.5 (Figure 2).

**Relation of RTL toclinico-pathological features, risk categories and response to induction chemotherapy**

**Figure 3:**
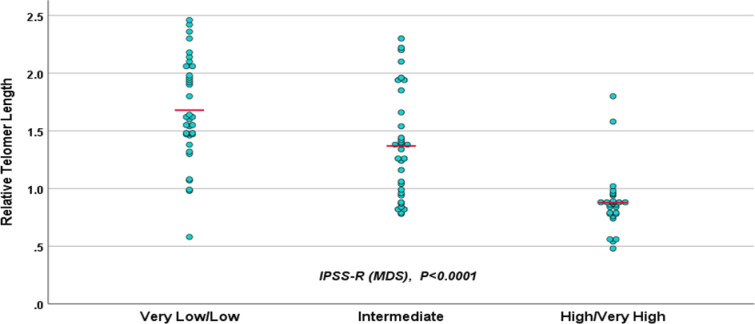
RTL is significantly reduced in relation to higher IPSS-R scoring system

**Figure 4:**
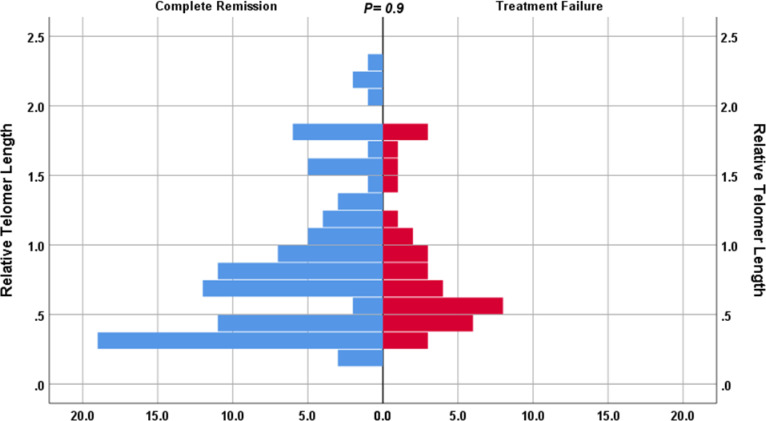
The distribution of RTL in AML cases showed no significant relation to complete remission rate

In MDS cases, shorter RTL was detected in MDS cases with pancytopenia (p < 0.0001), high bone marrow blast percentage (p <0.0001), poor risk karyotype (p < 0.0001) and high, very high R-IPSS (p < 0.0001; Figure 3). However, in AML cases, there was no significant relationship linking RTL to cytogenetic risk (p = 0.14). Also, the RTL showed no significant relation to the complete remission rate in AML cases (p = 0.9; Figure 4).

**The association between RTL and overall survival outcome in AML&MDS cases**

**Figure 5:**
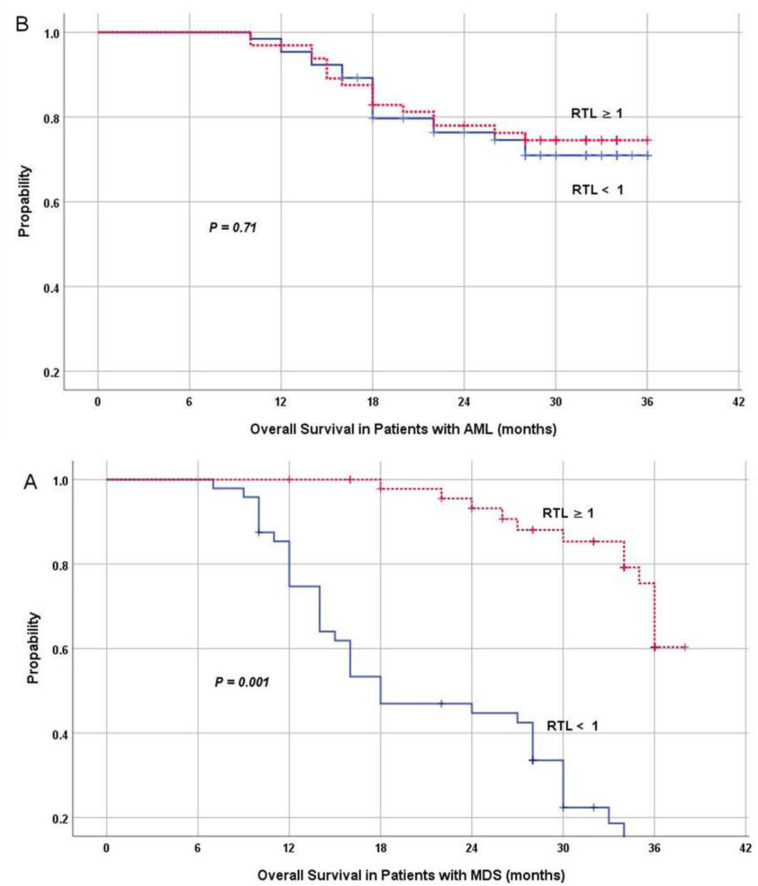
Short RTL was associated with inferior OS in MDS patients (A); no impact on OS in AML (B)

In MDS cases, univariate analysis revealed that age ≥ 60 years, high IPSS-R and shorter RTL (<1, median cut-off) were associated with inferior overall survival p=0.007 (Table2 and Figure.5). Multivariate analysis revealed that short telomere length was independently associated with adverse prognosis for survival in MDS HR 3.42 (Confidence interval 1.46-7.93, p 0.004), along with revised international prognostic scoring system (Table 2). While in AML cases, RTL did not affect the overall survival (Figure. 5).

**Table 2:** Univariate and multivariate regression analysis of risk factors for overall survival in MDS

	**Univariate**			**Multivariate**		
	HR	95% CI	p	HR	95% CI	p
Sex (male)	1.6	0.7 – 4.2	0.12			
Age (≥ 60 years)	1.82	1.12- 3.22	**0.03**	1.55	0.73 - 3.27	0.25
IPSS-R						0.0001
IPSS-R (Intermediate)	7.18	3.52 - 14.39	0.001	5.09	1.76 - 14.65	0.003
IPSS-R (High/Very High)	12.22	9.44 - 16.28	0.0001	11.39	8.97 - 19.35	0.0001
RTL	3.95	1.88 - 7.62	0.0001	3.42	1.46 - 7.93	0.004

HR (hazard-ratio), CI (confidence-interval), IPSS-R (revised-international prognostic scoring system), RTL (Relative Telomer Length)

## Discussion

 Cumulative knowledge from previous studies had hyopthesisd that altered telomere homeostasis plays a possible role in bone marrow failures, leukemias and other malignancies^   17 ^. Telomere erosion in tumor cells was found to predict worse prognosis with more advanced disease in chronic lymphocytic leukemia  ^18^^, ^^19^, myeloma^20^ and various cancers ^        20 ^. Mendelian short telomere is sufficient to promote premature age-related clonal hematopoiesis, primarily associated with MDS and AML^   21 ^. Therefore, we examined the relationship between RTL and clinical outcome in MDS and AML. 

In the current study, MDS cases (median age 55 years; 37-76) were older than AML cases (median age 44 years; 17-67). RTL was significantly longer in the MDS versus AML cases (p = 0.03). MDS cases showed significant reduction in RTL with increasing age at diagnosis (p < 0.0001), but AML cases showed positive insignificant correlation. This finding contradicts with the phenomenon that occurs normally with aging ^22^^,^^23^. Similar findings were evident in the study done by Hosnijeh, Matullo ^24^ on B-cell lymphoma patients and Xie Wu^25^ on soft tissue sarcomas patients. Dagg Pickett^26^ hypothesized that RTL elongation is caused by a defective trimming of telomeres during embryogenesis, and unbalanced telomerase activity. It has been hypothesized that longer telomeres may increase cancer risk by allowing multiple cell divisions and deferring senescence and apoptosis, which allows the cells to assemble genetic alterations leading to cancer development^   27 ^. Also Rode Nordestgaard^28^  concluded that long telomeres are characterized by increased cancer risk, especially melanoma and lung cancer.

MDS is characterized by ineffective hematopoiesis resulting in peripheral cytopenias despite hypercellular bone marrow with increased risk of AML transformation^        29 ^. One of possible explanation is telomere shortening which allows cells to be more susceptible to chromosomal instability and cell division arrest. It is assumed that the lack of telomerase activity in MDS results in failure of cells to overcome replicative senescence^        30 ^. The reason why telomerase is not upregulated in MDS is unknown, and studies have failed to show any responsible acquired telomerase-regulated genetic abnormalities in this patient group, although there are several germline mutations described in TERC and TERT predisposing to MDS/AML ^31^^, ^^32^ ^.^

Our study revealed a significant impact of short telomere length on the number of cytopenias in MDS cases. Williams Heppel ^12^ showed that shorter telomere length was associated with increased number of cytopenia and our data showed significant negative correlation between RTL and blast cell count which were similar to Gohring Lange ^33^ who demonstrated that MDS associated with shorter tolemeres was more prone to leukemic transformation.

In fact, our data demonstrated that shorter RTL group (< 1.3; median cut-off) was associated with inferior OS (p=0.007) and that was significantly associated with poor prognostic parameters as increased number of cytopenia, increased blast count, poor cytogetic risk group and high/very high R-IPSS risk group, similar to Ohyashiki Iwama^34^ who reported that MDS patients with short telomere at the time of diagnosis had a high incidence of complex chromosome abnormalities, rapid disease progression and shorter survival time. Furthermore, short telomere caused end-to-end fusion that led to genetic instability with induced leukemogenesis^   35 ^. Also, Hwang Kim^36^ concluded that short telomeres determined the cell fate and poor survival was attributed to tumor burden. 

Telomere loss is believed to limit the growth of many somatic stem cells, thereby acts as tumor suppressor mechanism^   7 ^. Unlike MDS cells, AML cells showed evidence of upregulated telomerase activity that allows blast cells to continue to replicate despite accelerated telomere shortening during leukaemogesnesis^37^^.^ It was also explained in experimental models of AML and demonstrated that acute leukemia-causing fusion genes *MLL-AF4* and *AML1-ETO* have been reported to upregulate *TERT* expression^        38 ^.

Our results showed positive insignificant relation between RTL and age in AML patients. Also, no relation with cytogentic risk group (p = 0.14) unlike the finding by Capraro Zane^39^ who demonstrated that shorter telomeres length in AML patients was associated with complex cytogenetics. While Watts Dumitriu ^40^ showed longer TL was associated with a commonly mutated DNA modifying enzymes (*IDH1/2, DNMT3A, TET2)*, mutations in *FLT3* and other signaling mutations were associated with shorter TL.

Shortened telomere length and increased telomerase activity were associated with chemotherapy resistance, rapid disease progression and dismal prognosis in patients with acute leukemia^   41 ^. However, in a recent report, telomere length was not associated with any prognostic information in patients with acute myeloid leukemia and high-risk myelodysplastic syndrome^        42 ^. Our data showed no relation between RTL with response rate (p = 0.9) and survival (p = 0.7). This could be explained by different patients study, protocol therapy. Multivariate regression analysis revealed that short telomere length was independent prognostic marker for survival in MDS (p 0.004) HR 3.42, CI 1.46-7.93, along with revised international prognostic scoring system mainly intermediate, high, very high.

Finally, long-standing clinical observations indicate that short dysfunctional telomeres may result in chromosomal instability and clonal evolution, resulting in dreadful clinical consequences in different malignant subtypes. Our results also document that telomere dysfunction plays a major role in MDS biology and progression to AML, that is why therapeutic targets as telomerase inhibitors worth more studies in myeloid disorders.
